# Supplementary material for: Comparative Profiling of Hot and Cold Brew Coffee Flavor Using Chromatographic and Sensory Approaches
Source: Foods. 2022 Sep 22;11(19):2968. doi: 10.3390/foods11192968 (PMC9562860; doi:10.3390/foods11192968)
Supplement: Supplementary file 1 [file foods-11-02968-s001.zip › foods-1895011-supplementary.pdf]

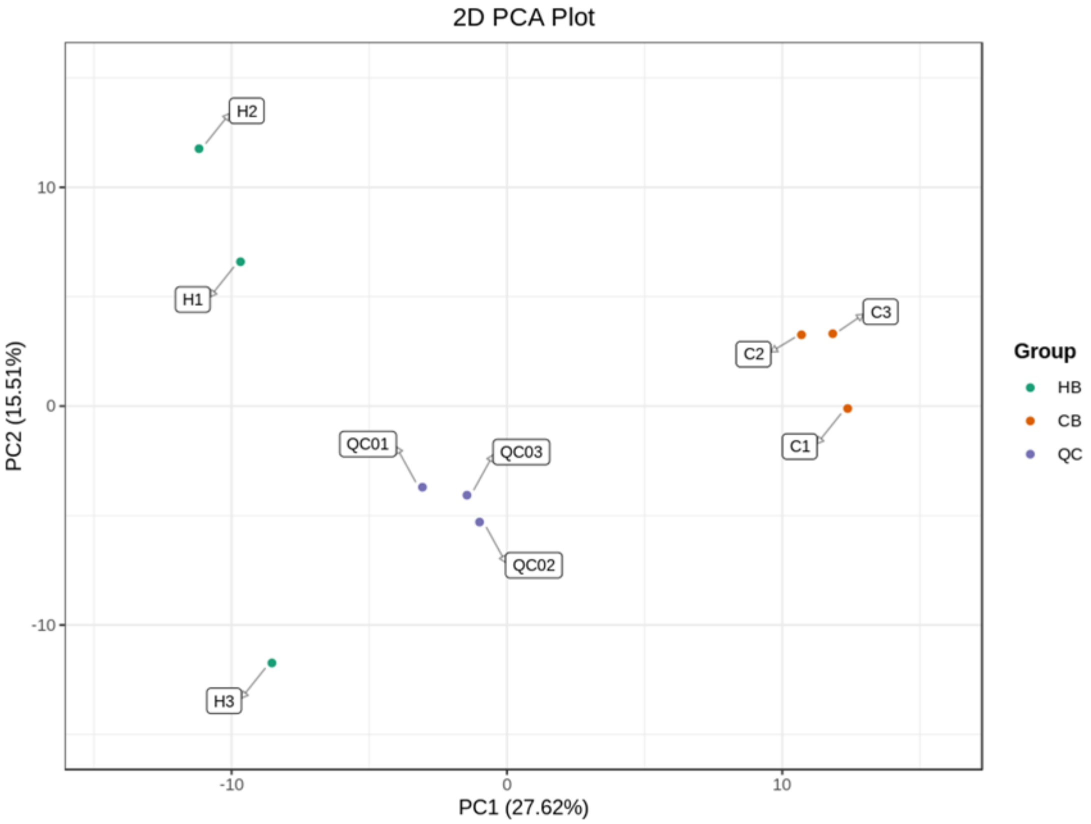

Figure S1. Principal component analysis (PCA) of the overall non-volatiles in hot and cold brew coffee using ultra-performance liquid chromatography-mass spectrometry/mass spectrometry (UPLC-MS/MS). HB: hot brew coffee; CB: cold brew coffee; QC: quality controls.

Table S1. Hot and cold brew coffee sensory attributes and their 5-point scale reference for panel training.

Term	Definition	Reference
Color	The visual perception of the coffee product	Green tea =1，Coke =5
Nutty	The nonspecific nutlike flavors that are characteristic of several different nuts, for example, peanuts, hazelnuts, pecans, and almonds [5]	Roasted mixed nuts =5
Coffee	The perception of robust coffee flavor that is rounded with body [21]	Italian blended coffee beans =5
Fruity	A sweet, floral aromatic blend of a variety of ripe fruits	Freshly cut lemon zest=5
Sweet	Fundamental taste sensation elicited by sugars	White granulated sugar =5
Sour	Fundamental taste sensation elicited by citric acid	Freshly cut lemon=5
Bitter	Fundamental taste sensation elicited by caffeine and quinine	100% dark chocolate =5
Astringent	Chemical feeling factor on the tongue or oral cavity described as puckering or dry	Dry red wine=4
